# Effect of whole body vibration therapy in the rat model of steroid-induced osteonecrosis of the femoral head

**DOI:** 10.3389/fcell.2023.1251634

**Published:** 2023-10-09

**Authors:** Jia-Qing Tian, Teng-Fei Wei, Yu-Rou Wei, Fang-Jun Xiao, Xian-Shun He, Kun Lin, Shun Lu, Xiao-Ming He, Wei He, Qiu-Shi Wei, Xiao-Wei Xiang, Min-Cong He

**Affiliations:** ^1^ The Third Clinical Medical College, Guangzhou University of Chinese Medicine, Guangzhou, Guangdong, China; ^2^ The Third Affiliated Hospital of Guangzhou University of Chinese Medicine, Guangzhou, Guangdong, China; ^3^ Guangdong Research Institute for Orthopedics and Traumatology of Chinese Medicine, Guangzhou, Guangdong, China; ^4^ Shenzhen Luohu Traditional Chinese Medicine Hospital, Shenzhen, Guangdong, China

**Keywords:** steroid-induced osteonecrosis of the femoral head, whole body vibration therapy, mechanical stimulation, blood flow supply, osteogenic differentiation; H-vessels

## Abstract

**Background:** Steroid-induced Osteonecrosis of the Femoral Head (SIONFH) is a skeletal disease with a high incidence and a poor prognosis. Whole body vibration therapy (WBVT), a new type of physical training, is known to promote bone formation. However, it remains unclear whether WBVT has a therapeutic effect on SIONFH.

**Materials and methods:** Thirty adult male and female Sprague-Dawley (SD) rats were selected and randomly assigned to three experimental groups: the control group, the model group, and the mechanical vibration group, respectively. SIONFH induction was achieved through the combined administration of lipopolysaccharides (LPS) and methylprednisolone sodium succinate for injection (MPS). The femoral head samples underwent hematoxylin and eosin (H&E) staining to visualize tissue structures. Structural parameters of the region of interest (ROI) were compared using Micro-CT analysis. Immunohistochemistry was employed to assess the expression levels of Piezo1, BMP2, RUNX2, HIF-1, VEGF, CD31, while immunofluorescence was used to examine CD31 and Emcn expression levels.

**Results:** The H&E staining results revealed a notable improvement in the ratio of empty lacuna in various groups following WBVT intervention. Immunohistochemical analysis showed that the expression levels of Piezo1, BMP2, RUNX2, HIF-1, VEGF, and CD31 in the WBVT group exhibited significant differences when compared to the Model group (*p* < 0.05). Additionally, immunofluorescence analysis demonstrated statistically significant differences in CD31 and Emcn expression levels between the WBVT group and the Model group (*p* < 0.05).

**Conclusion:** WBVT upregulates Piezo1 to promote osteogenic differentiation, potentially by enhancing the HIF-1α/VEGF axis and regulating H-vessel angiogenesis through the activation of the Piezo1 ion channel. This mechanism may lead to improved blood flow supply and enhanced osteogenic differentiation within the femoral head.

## 1 Introduction

Steroid-induced Osteonecrosis of the Femoral Head (SIONFH) is a skeletal disease with a high incidence and a poor prognosis (LIANG et al., 2023). Its debilitating effects could eventually lead to articular cartilage collapse and subsequent osteoarthritis (CAO et al., 2016). High doses of glucocorticoids are recognized to be major risk factors of SIONFH (IKEUCHI et al., 2015). Over the past decades, glucocorticoids have been widely used as effective immunosuppressive and anti-inflammatory medications in clinical practice. However, numerous studies have demonstrated that prolonged or excessive use of glucocorticoids could lead to the destruction of the femoral head’s internal blood supply and disrupt local nutrition. (MARSTON et al., 2002; YAO et al., 2020). This leads to the apoptosis of bone cells and the subsequent necrosis of bone tissue (LIU et al., 2022a). Accordingly, it is crucial and essential to comprehend the exact pathological mechanisms and develop effective treatments in order to disrupt the process of SIONFH.

Whole body vibration therapy (WBVT) training is gaining significant attention as a potential treatment for low levels of bone mass. The effectiveness of WBVT in treating osteoporosis has been reported in several studies (KOSTYSHYN et al., 2022a; LIU et al., 2022b). Our previous research established that WBVT can reduce osteoporosis by increasing BMD, bone architecture and bone strength (WEI et al., 2015). There is growing evidence that mechanical stimuli can be used to direct stem cell differentiation towards a variety of different tissue lineages (KELLY et al., 2010; LI et al., 2011). For instance, it has been shown that applying compression (SITTICHOKECHAIWUT et al., 2010) or tension (SARRAF et al., 2011)can promote osteogenic differentiation in mesenchymal stem cells (MSCs). Other work has shown that mechanical stimulation can promote bone growth by activating Piezo1 ion channels (JIANG et al., 2021a).

Piezo1 protein is a membrane-bound trimeric Ca^2+^ channel protein that was initially discovered in 2006 (PRISBY et al., 2008). Because of its unique structure, it can cling to the cell membrane’s surface and control the flow of ions inside and outside the membrane by sensing mechanical stress stimuli. This allows the sensed stress stimuli to be transformed into electrical impulses for further transmission (ZENG et al., 2022). *In vitro* experiment examining femoral head samples, we found that Piezo1 expressed differently depending on the location within the femoral head (TENGFEI et al., 2023). It is discovered that Piezo1, whose expression is upregulated, encourages osteoblast development and postpones bone resorption (WU et al., 2021). In addition, it has been found that Piezo1 can be expressed in vascular endothelial cells (JIANG et al., 2021b; EMIG et al., 2021). It can sense changes in shear stress caused by blood flow, thus mediating the conduction of Na^2+^ and Ca^2+^ and regulating vascular tone and vascular development (BEECH, 2018; FANG et al., 2021; SWAIN et al., 2021).

Therefore, we conducted this study to find out if WBVT may treat SIONFH by encouraging the expression of Piezo1.

## 2 Materials and methods

All animal experimental procedures were approved by the Animal Care and Use Committee of the Animal Center of Guangzhou University of Chinese Medicine (No. 20220111007).

### 2.1 Reagents and antibodies

Methylprednisolone was obtained from The First Affiliated Hospital of Guangzhou University of Chinese Medicine (registration number:H20170199, Pfizer Manufacturing Belgium Nv); Lipopolysaccharides (LPS) was obtained from Solarbio (Beijing, China,L8880). Anti-Piezo1 Antibody (DF12083), anti-HIF-1α Antibody (AF1009), anti-VEGF Antibody (AF5131); anti-RUNX2 Antibody (AF5186) anti-BMP2 Antibody (AF5163) and secondary antibodies were obtained from Affinity (Jiangsu Province, China). Anti-Endomucin (SC-65495) was obtained from Santa Cruz (Paso Robles, CA, United States). Anti-CD31 was obtained from invitrogen (WD3255103, Waltham, MA, United States). Goat Anti-Rabbit IgG/SAlexa Fluor 488 (K0034G-AF488) and goat Anti-Rat IgG/SAlexa Fluor 594 (K0034G-AF488) were obtained from Solarbio (Beijing, China).

### 2.2 Animals and experimental grouping

Thirty 3-month-old specific pathogen-free (SPF) Sprague-Dawley (SD) rats were used in this study. The rats were purchased from the Animal Center of Guangzhou University of Chinese Medicine (SCXK 2018-0034, Guangzhou, China). Male rats weighed approximately 300 ± 50 g, while female rats weighed approximately 250 ± 50 g. The rats were divided randomly into three groups: Control group (n = 10), Model group (n = 10), and WBVT group (n = 10). All rats were housed under standardized laboratory conditions with the same temperature and humidity. They were provided with unlimited access to water and a standard diet.

### 2.3 Establishment of the SIONFH model group, control group and WBVT-treated group

The SIONFH model was constructed following these steps. Before drug dosing, the rats were weighed. For the first 3 days, a daily intraperitoneal (i.p.) injection of LPS (10 ug/kg, Sigma-Aldrich) was administered. Subsequently, an intramuscular (i.m.) dose of methylprednisolone (MP, 60 mg/kg, Pfizer) was given weekly for the next 4 weeks. MP was alternately injected into the left and right gluteus muscles. Starting from the second day after MP injection, the WBVT group received WBVT sessions twice daily for 10 min each, with a 5-min rest in between (Vibration frequency: 30 Hz, acceleration: 0.2 g) for 4 consecutive weeks. After 4 weeks of MP medication, the animals were sacrificed, and femoral heads were collected for further analysis.

### 2.4 Micro CT analysis

The microarchitecture of the femoral head’s trabecular bone was assessed using a high-resolution Micro CT (NEMO Micro CT, PingSeng Technology, China). After removing soft tissue, trabecular bone from the metaphyseal regions of the femoral head was analyzed using the Micro CT machine. Measurements, including Bone Volume Fraction (BV/TV), Bone Trabecular Number (Tb.N), Bone Trabecular Thickness (Tb.Th), and Bone Trabecular Separation (Tb.Sp), were obtained. The region of interest (ROI) was specifically defined as the subcortical weight-bearing area of the femoral head.

### 2.5 Histological staining

The femoral bone samples were fixed in 10% paraformaldehyde solution for 48 h and then decalcified using 10% EDTA solution for 4 weeks, with the solution changed every 2 days. Subsequently, some specimens from each group were embedded in paraffin, and 4 μm sections were obtained in the coronary position. The sections were then dewaxed in xylene, rehydrated in graded ethanol, and the residual water was removed using distilled water for HE staining. HE staining was used to observe the structural alterations of the femoral head, as well as to assess the femoral head’s trabecular structure and the ratio of empty lacuna.

### 2.6 Immunohistochemical staining

Immunohistochemistry was performed following standard procedures. Four-micron-thick tissue sections were baked in a 60°C oven for 30 min, deparaffinized in xylene, and rehydrated in a descending series of ethanol in distilled water for 2 min. After quenching endogenous peroxidase activity and blocking non-specific binding, the specimens were incubated overnight at 4°C with the following primary antibodies: anti-Piezo1 (1:100; Affinity), anti-RUNX2 (1:100; Affinity), anti-BMP2 (1:100; Affinity), anti-CD31 (1:200; Abcam), anti-VEGF (1:100; Affinity), and anti-HIF-1α (1:100; Affinity). The slices were then incubated with secondary antibodies for 2 h at 37°C. For visual analysis, the product was stained with diaminobenzidine (DAB) and counterstained with Mayer’s hematoxylin. Finally, these slices were examined under an electron microscope for protein expression analysis.

### 2.7 Immunofluorescence staining

The other specimens were dewaxed in xylene for immunofluorescence staining. Then, these slices were incubated with normal goat serum for 2 h to block nonspecific antibody interference and subsequently incubated overnight at 4°C with anti-CD31 (1:200; Abcam) and anti-Emcn (1:100; SANTA) primary antibodies. The slices were then incubated with secondary antibodies, either Goat Anti-Rabbit IgG/SAlexa Fluor 488 (Solarbio, 1:100) or Goat Anti-Rat IgG/SAlexa Fluor 594 (Solarbio, 1:100), for 2 h at 37°C. Next, these slices were counterstained with the nuclear marker 4,6-diamino-2-phenylindole (DAPI). All images were observed using a fluorescence microscope (Olympus).

### 2.8 Statistical analysis

The statistical analyses were performed using IBM SPSS Statistics 20 software. The results were presented as mean ± standard deviation, and differences with *p* values <0.05 were considered statistically significant. Data that satisfied the tests for normal distribution and homogeneity of variance were analyzed using ANOVA for multiple comparisons, while data that did not meet these assumptions were analyzed using nonparametric tests.

## 3 Results

### 3.1 WBVT reduces the bone destruction effect of SIONFH

**FIGURE 1:**
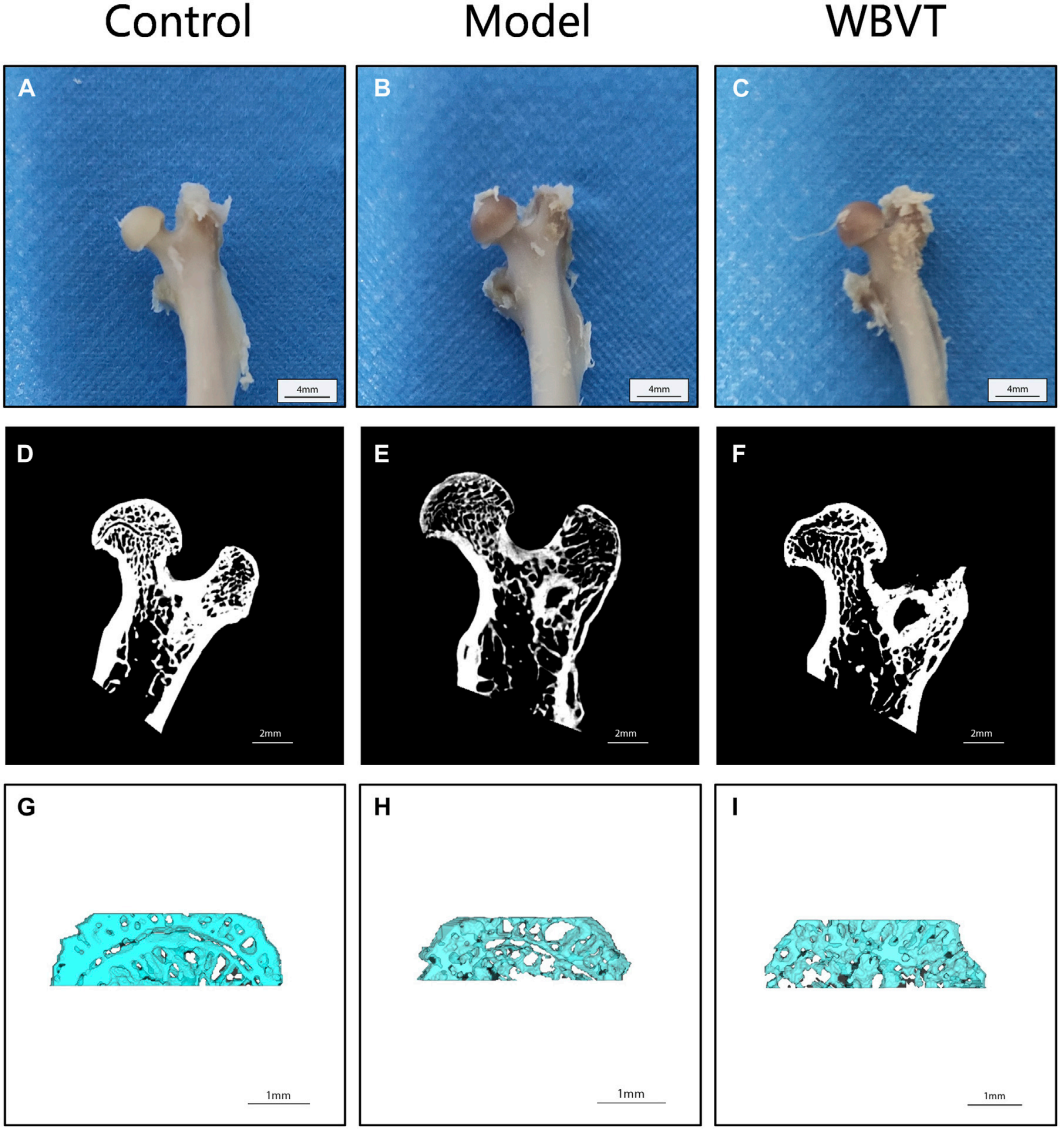
Micro CT results reveal the femoral bones of rats in different groups. In **(A, D, and G)**, the images display the femoral bones of rats in the blank group. **(B, E, and H)** show the femoral bones of rats in the SIONFH group, while **(C, F, and I)** depict the femoral bones of SIONFH rats after WBVT treatment.

**FIGURE 2:**
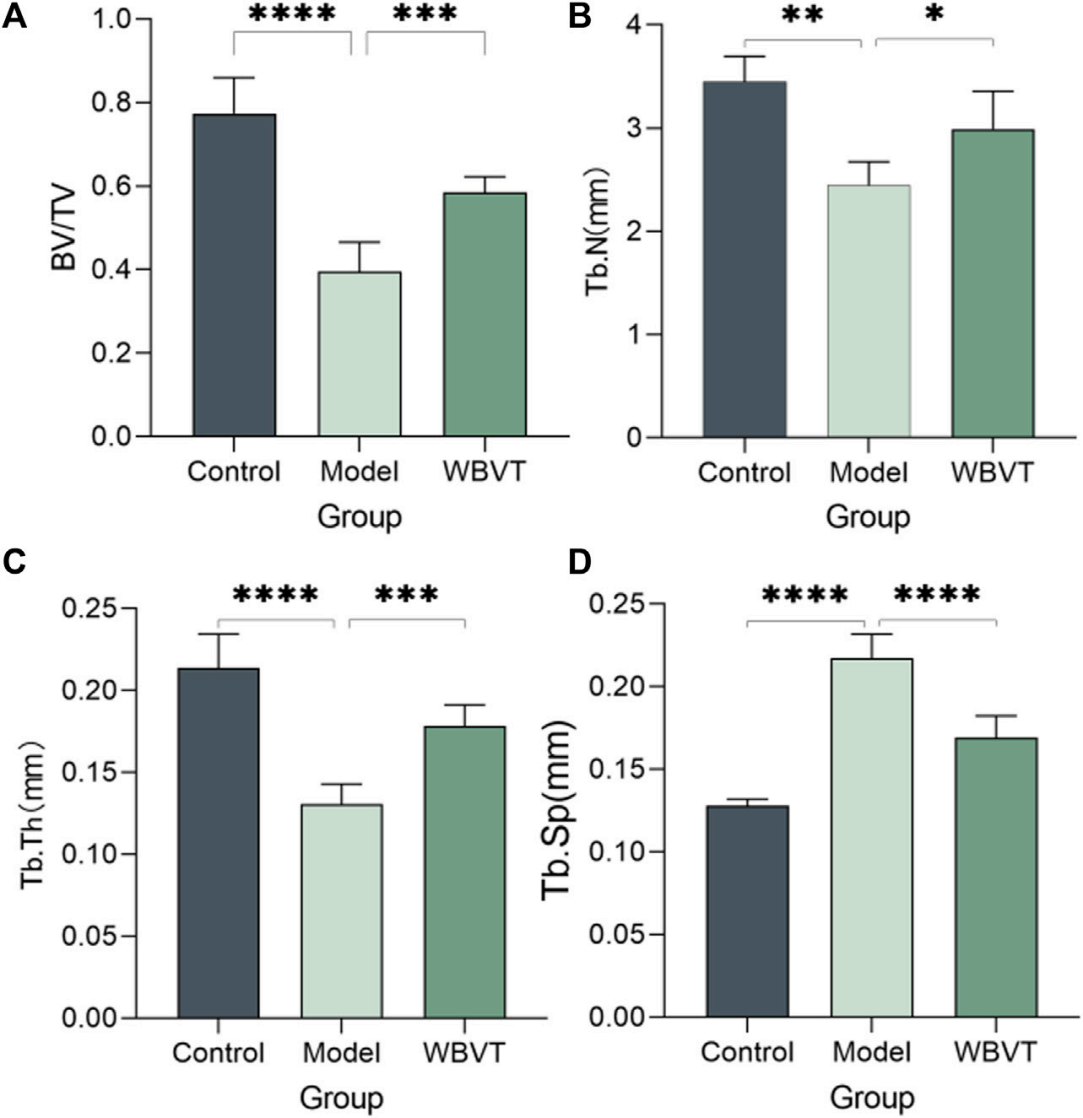
Micro CT quantitative results, **(A)** shows the bone volume fraction; **(B)** shows the number of bone trabeculae; **(C)** shows the thickness of bone trabeculae; **(D)** shows the separation of bone trabeculae, which was found to be statistically significantly different (*p* < 0.05) after statistical analysis of the three groups.

As shown in Figures 1, 2, Micro CT scans confirmed the successful construction of the SIONFH rat model in the experimental group. In comparison to the Control group, the femoral head of rats in the SIONFH group exhibited surface structure destruction, resulting in a rough appearance. Additionally, numerous cavities were observed within the cut femoral head, indicating the impact of methylprednisolone on the development of SIONFH. Compared with the blank group, the rats in the SIONFH group showed a reduction in bone density and bone parameters BV and BV/TV. However, after whole body mechanical vibration therapy, improvements were observed in the roughness of the femoral head surface, and the number of cavities inside the femoral head decreased when compared to the model group, as depicted in Figure 1. Additionally, our observations indicated that gender did not have a significant impact on the efficacy of WBVT treatment. The quantitative analysis of Micro CT parameters demonstrated that WBVT had a therapeutic effect on early steroid-induced Osteonecrosis of the Femoral Head. Specifically, the femoral head trabeculae, especially in the subchondral region, showed significant improvement, as depicted in Figure 2.

### 3.2 HE staining to assess the curative efficacy of WBVT for SIONFH

**FIGURE 3:**
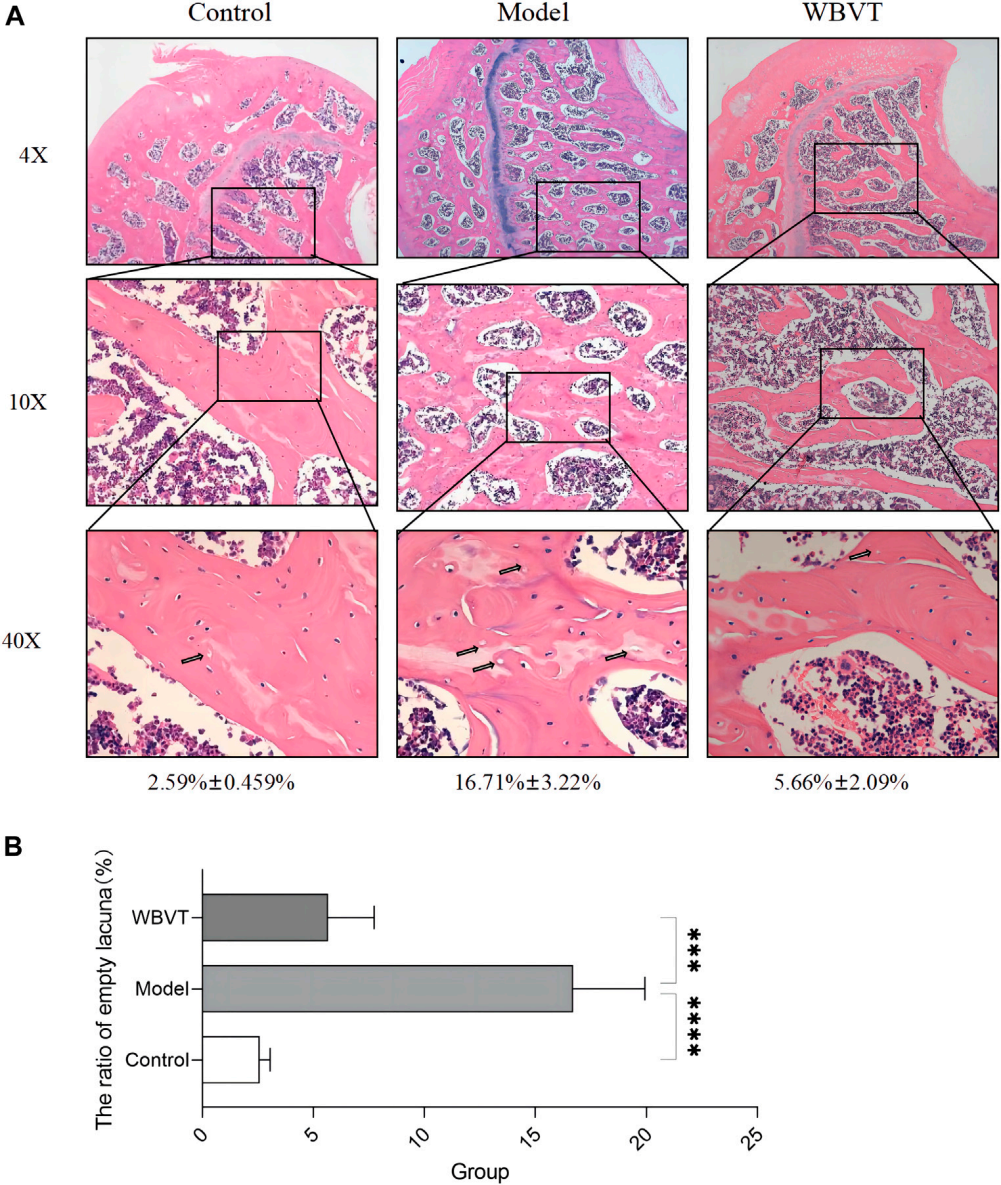
The results of HE staining. **(A)**: Compared to the Control group, the model group exhibited a noteworthy occurrence of osteoblast apoptosis, along with sparse bone trabeculae and interrupted continuity within the femoral head. However, these detrimental effects exhibited significant improvement with WBVT treatment. **(B)**: The ratio of empty lacuna within the femoral head at various groups.

The Model group exhibited significant differences compared to the blank group, including a considerable area of missing trabeculae within the femoral head, sparse trabeculae, interrupted continuity, extensive apoptosis of osteocytes, and a higher number of empty bone traps. However, following WBVT treatment, notable improvements were observed. The trabeculae became denser and more orderly arranged, with fewer defects, enhanced trabecular thickness, and a reduced number of empty bone lacunae, as depicted in Figure 3. It is noteworthy to mention that the results did not indicate significant gender-based differences among rats within the same group.

### 3.3 WBVT promotes the expression of RUNX2, BMP2, CD31, HIF-a, and VEGF by promoting the expression of Piezo1

**FIGURE 4:**
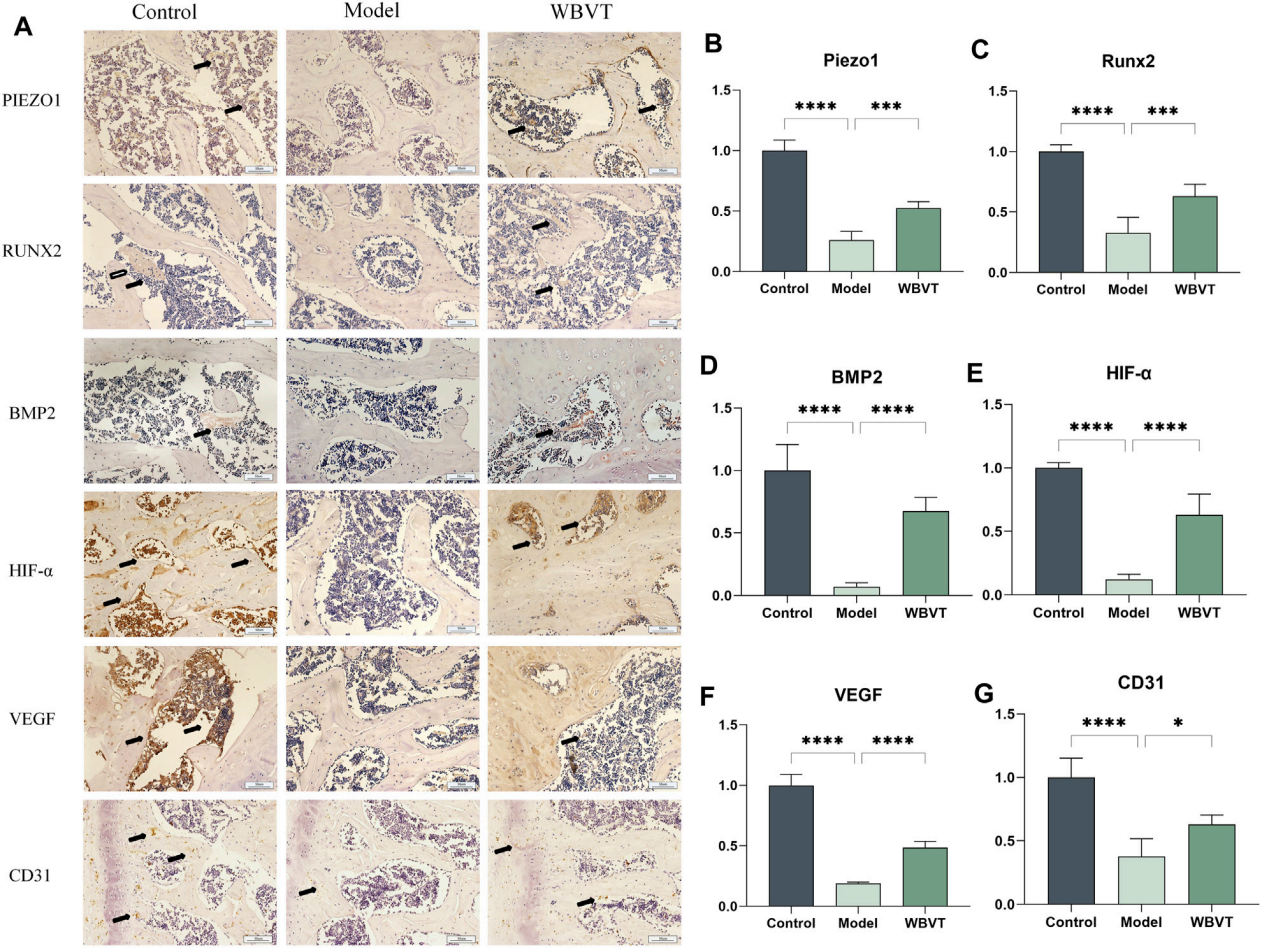
In **(A)**, the immunohistochemical results of Piezo1, RUNX2, BMP2, CD31, HIF-a, and VEGF are presented. Additionally, **(B–G)** display the corresponding quantitative immunohistochemical results. In comparison to the model group, both the blank and WBVT groups exhibited significantly higher expression levels of Piezo1, RUNX2, BMP2, CD31, HIF-a, and VEGF. These differences were statistically significant (*p* < 0.05).

The immunohistochemical and quantitative results revealed that WBVT effectively enhanced the expression of several key factors within the femoral head. Figure 4 illustrates the increased expression levels of Piezo1, RUNX2, BMP2, CD31, VEGF, and HIF-a following the WBVT treatment.

### 3.4 Expression levels of H-vessels in the rat femoral head

**FIGURE 5:**
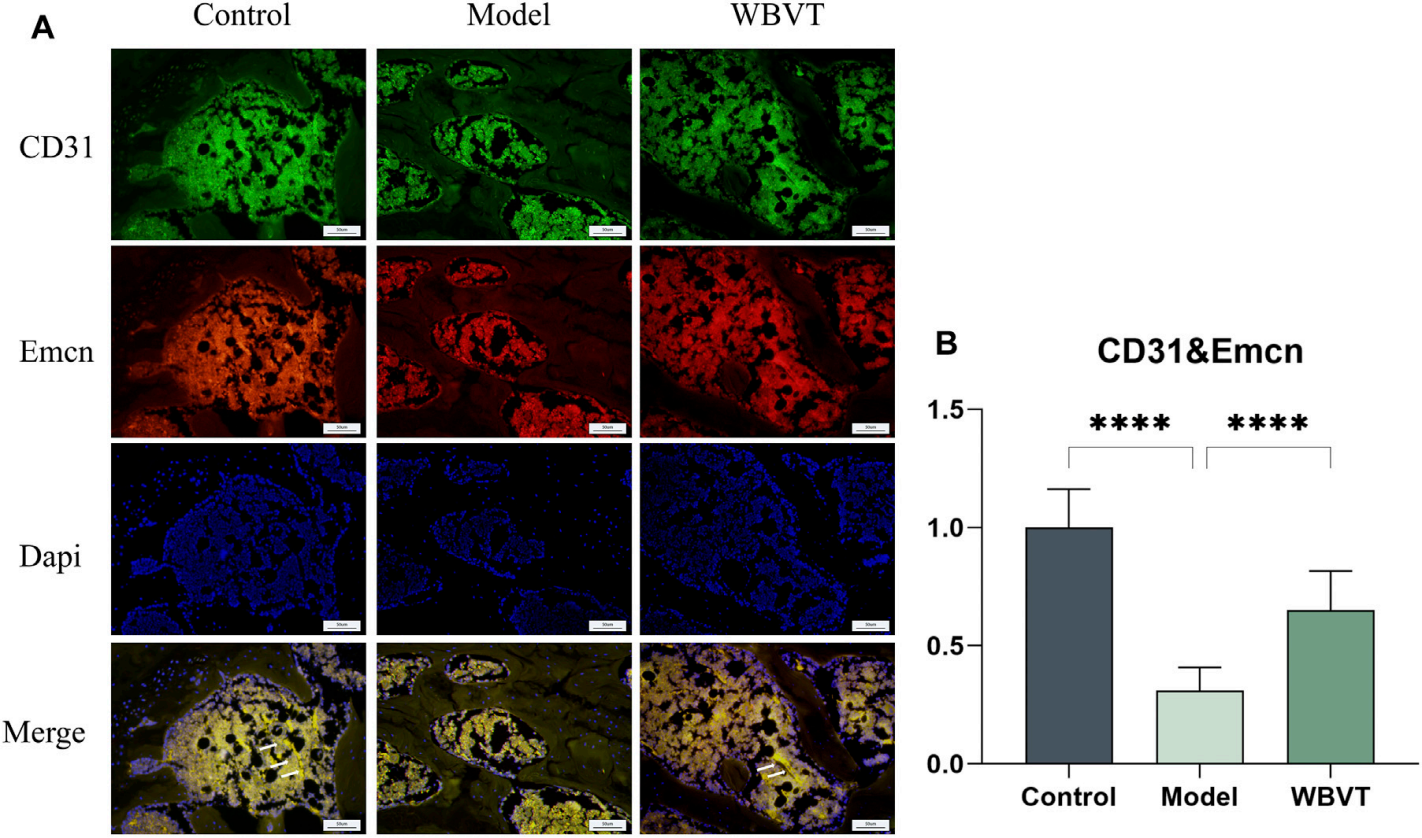
**(A)** shows the expression of CD31 and Emcn in the rat femoral head. **(B)** shows the quantitative Immunofluorescence results. Model group was significantly lower than that in the blank and WBVT groups, and both were statistically significant (*p* < 0.05)

CD31 and Emcn were identified as specific markers for H-vessels vascular endothelial cells, and the vessels co-localized with both CD31 and Emcn, as depicted in Figure 5. The results demonstrated that in the femoral head of rats, the expression of H-vessels in the Model group was significantly lower compared to the normal group. However, following the intervention of WBVT, the expression of H-vessels noticeably increased, with a statistically significant difference observed.

## 4 Discussion

In this study, we investigated the therapeutic effects of WBVT on SIONFH rats, analyzing the HE pathological staining and Micro CT results from the blank group, Model group, and WBVT group. Our findings revealed that WBVT effectively treated SIONFH in rats, likely through its positive impact on blood flow supply in the femoral head and promotion of osteoblastogenesis. Notably, the expression level of Piezo1 in the femoral head of WBVT rats exhibited a significant increase compared to the Model group, indicating that Piezo1 may play a key role in mediating the stress stimulation induced by WBVT, thereby facilitating SIONFH self-repairing.

The significance of biomechanical stimulation in enhancing bone strength has been widely acknowledged for a considerable period (FERRERI et al., 2011; CHAN et al., 2013; HU et al., 2014). During mechanical loading, bone marrow mesenchymal stem cells (BMSC) sense fluid flow in the lacunar-canalicular system and promote osteogenic gene expression, modify BMSC morphology, volume, and cytoskeleton structure, and drive BMSC proliferation and differentiation (WANG et al., 2022). Numerous studies have explored the use of mechanical stress stimulation as a potential treatment for osteoporosis (KOSTYSHYN et al., 2022b; FAN et al., 2023). In a prior study conducted by the authors' group, it was discovered that WBVT can effectively promote bone trabeculation, enhance osteoblast osteodifferentiation, and improve bone strength in osteoporotic rats (WEI et al., 2015). The effect of mechanical stimulation on bone tissue depends on many factors, including vibration frequency, vibration amplitude, and duration. A particular study revealed that (BECK et al., 2010), WBVT conducted twice a week has been shown to effectively reduce bone loss in the hip and spine while also increasing muscle strength in the lower extremities. Furthermore, in postmenopausal women, combining strength training with WBVT can lead to improvements in bone density. From a microscopic perspective, WBVT has demonstrated the ability to enhance bone microstructure and tissue mechanical properties by promoting bone anabolic reactions (WUERMSER et al., 2015; JING et al., 2018).

However, the specific mechanism by which WBVT causes mechanical stress to stimulate bone formation is not fully understood. And the key to affecting bone formation is the ability to mediate osteoblast lifespan and inhibit osteoclast differentiation (PRISBY et al., 2008; PASQUALINI et al., 2013; XIE et al., 2016). The mechanosensitive ion channel Piezo1 is a mechanically stimulated, non-selective cation channel that converts mechanical stress into electrochemical signals for further conduction (DOUGUET et al., 2019). It is essential for bone formation and regulation of bone resorption in postnatal mice (SUN et al., 2019). And with the increase of mechanical stress after birth, it has been found that the expression of Piezo1 gradually increases in mice (ZHOU et al., 2020). Our research further proved that WBVT exerts a treatment effect via promoting the expression of the Piezo1 protein in SIONFH. SUGIMOTO et al. reported that activation of the Piezo1 protein channel enhances the production of BMP2, thereby accelerating osteoblast formation while inhibiting the differentiation of mesenchymal stem cells into adipocytes (SUGIMOTO et al., 2017). SONG et al. conducted *in vitro* experiments and found that elevated Piezo1 expression induces the expression of the Runx-2 gene in osteoblasts (SONG et al., 2020). In human bone marrow mesenchymal stem cells, the silencing of Piezo1 using lentivirus resulted in a significant decrease in the expression of proteins associated with osteogenesis, such as RUNX2, OPN, and Osteocalcin. This indicates that inhibiting Piezo1 hinders the differentiation of human bone marrow mesenchymal stem cells into osteoblasts (SUN et al., 2019). Our research findings align with the aforementioned conclusions. We observed that the protein expressions of osteogenesis-related markers, including BMP2 and RUNX2, were upregulated following the treatment of WBVT. Based on these results, we can confidently conclude that WBVT has the potential to promote osteogenic differentiation through the activation of the Piezo1 protein.

Blood flow disruption is one of important causes of SIONFH. And current research have not yet shown whether vibration mediates Piezo1 expression to impact angiogenesis. When researchers knocked out Piezo1 on mouse endothelial cells, they observed that the mice failed to form blood vessels during embryonic development, resulting in embryonic death (XU et al., 2022). In contrast, in a mouse hindlimb ischemia model, normal mice had significantly better recovery of blood flow after ischemia than Piezo1 knockout mice (KANG et al., 2019). The above findings suggest that Piezo1 expression is vital in providing adequate nutrition for blood vessels, promoting structural reorganization of damaged blood vessels, and playing a crucial role in regulating vascular structure and embryonic development. Based on our results, we observed that the expression levels of HIF-a and VEGF at the femoral head of rats in the Model group were significantly lower compared to those in the blank group. However, following WBVT intervention, the levels of HIF-a and VEGF were improved. Additionally, significant neovascularization was observed in the blank group, as indicated by the MVD results. To quantitatively assess microvessel density, we utilized immunohistochemical staining techniques, specifically targeting markers of vascular endothelial cells, such as CD31 (NOWAK-SLIWINSKA et al., 2018). The model group of rats showed a considerable decrease in microvessel density compared to the control group. However, after the intervention of WBVT, there was a significant improvement in microvessel density. These findings strongly suggest that Piezo1 is capable of sensing the mechanical stress stimulation caused by WBVT, leading to an improvement in blood flow supply to the femoral head.

HIF-a plays a central role as the most important transcription factor family in regulating the cellular response to hypoxia (SEMENZA, 2012). It primarily influences the secretion of vasoactive factors like VEGF (Vascular Endothelial Growth Factor) by mediating the cellular perception of oxygen fluctuations, which in turn regulates the process of neovascularization. Recent studies have revealed an association between decreased expression of HIF-1a and VEGF and glucocorticoid-induced femoral head necrosis. Increasing the level of HIF-1a has been shown to facilitate the repair of SIONFH(WEINSTEIN et al., 2017). VEGF serves as a downstream target gene of HIF-1a and holds a critical role not only as the most significant regulator of vascular development but also as a key factor in angiogenesis, the process of forming new blood vessels (HOEBEN et al., 2004). Recent studies have found that (KUSUMBE et al., 2014), there is a coupling between H-vessels angiogenesis and osteogenesis, and previous studies have demonstrated a strong association with the onset and regression of osteoporosis. H-vessels is (KUSUMBE et al., 2014) recently discovered special vascular subtype, which is widely distributed in the epiphysis and femoral head, is mainly characterized by high expression of CD31/EMCN (called H-vessels vascular endothelial cells), which has a significant correlation with bone volume and bone strength (GAO, F, et al., 2020; WANG, L, et al., 2017). Simultaneously, H-vessels vascular endothelial cells are surrounded by a large number of osteoprogenitor cells, which promote bone formation by producing specific factors. Animal studies have found that (PENG, Y, et al., 2020), the development of osteoporosis is linked to a decrease in H-vessels. Meanwhile, some scholars have also isolated H-vessels vascular endothelial cells in the human femoral head (GAO et al., 2020). Based on this, we formulated the bold hypothesis that H-vessels angiogenesis could enhance blood supply to the femoral head and consequently facilitate the repair of femoral head necrosis. Subsequently, we conducted experiments to verify this hypothesis. In our study, the number of type H-vessels (CD31 and EMCN) was dramatically enhanced in the WBVT group with a striated distribution, but significantly decreased in the model group with sparse and punctate distribution. The results indicate that WBVT intervention can promote H-vessels angiogenesis. And Piezo1 is a key pathway for sensing WBVT stimulation, thus suggesting that WBVT regulates H-vessels angiogenesis probably through Piezo1 sensing mechanical stress stimulation, improving the cellular perception of oxygen changes and affecting the expression of HIF-1a/VEGF axis, thus improving blood flow supply at the femoral head and promoting osteogenic differentiation within the femoral head.

In conclusion, our study demonstrates for the first time that WBVT upregulates Piezo1 to promote osteogenic differentiation. Piezo1 appears to play a crucial role in transmitting stress stimuli and promoting osteogenic differentiation. Simultaneously, WBVT may activate the Piezo1 ion channel to promote the HIF-1a/VEGF axis, thereby regulating H-vessels angiogenesis, improving blood flow supply, and fostering osteogenic differentiation within the femoral head. Nevertheless, further research is necessary to fully understand the underlying mechanism.

WBVT promotes the HIF-1a/VEGF axis by influencing the expression of the Piezo1 pathway, which in turn regulates H-vessels angiogenesis, improves blood flow supply to the femoral head, and enhances osteogenic differentiation within the femoral head. Consequently, WBVT may effectively prevent the pathological process of hormonal femoral osteonecrosis.

## Data Availability

The raw data supporting the conclusion of this article will be made available by the authors, without undue reservation.
